# Dissociable Influences of Auditory Object *vs.* Spatial Attention on Visual System Oscillatory Activity

**DOI:** 10.1371/journal.pone.0038511

**Published:** 2012-06-05

**Authors:** Jyrki Ahveninen, Iiro P. Jääskeläinen, John W. Belliveau, Matti Hämäläinen, Fa-Hsuan Lin, Tommi Raij

**Affiliations:** 1 Athinoula A. Martinos Center for Biomedical Imaging, Department of Radiology, Massachusetts General Hospital, Harvard Medical School, Charlestown, Massachusetts, United States of America; 2 Harvard-MIT Division of Health Sciences and Technology, Cambridge, Massachusetts, United States of America; 3 Department of Biomedical Engineering and Computational Science (BECS), Aalto University, Espoo, Finland; 4 Institute of Biomedical Engineering, National Taiwan University, Taipei, Taiwan; Baycrest Hospital, Canada

## Abstract

Given that both auditory and visual systems have anatomically separate object identification (*“what”*) and spatial (*“where”*) pathways, it is of interest whether attention-driven cross-sensory modulations occur separately within these feature domains. Here, we investigated how auditory “what” *vs*. “where” attention tasks modulate activity in visual pathways using cortically constrained source estimates of magnetoencephalograpic (MEG) oscillatory activity. In the absence of visual stimuli or tasks, subjects were presented with a sequence of auditory-stimulus pairs and instructed to selectively attend to phonetic (*“what”*) *vs.* spatial (*“where”*) aspects of these sounds, or to listen passively. To investigate sustained modulatory effects, oscillatory power was estimated from time periods between sound-pair presentations. In comparison to attention to sound locations, phonetic auditory attention was associated with stronger alpha (7–13 Hz) power in several visual areas (primary visual cortex; lingual, fusiform, and inferior temporal gyri, lateral occipital cortex), as well as in higher-order visual/multisensory areas including lateral/medial parietal and retrosplenial cortices. Region-of-interest (ROI) analyses of dynamic changes, from which the sustained effects had been removed, suggested further power increases during Attend Phoneme *vs*. Location centered at the alpha range 400–600 ms after the onset of second sound of each stimulus pair. These results suggest distinct modulations of visual system oscillatory activity during auditory attention to sound object identity (“*what*”) *vs.* sound location (“*where*”). The alpha modulations could be interpreted to reflect enhanced crossmodal inhibition of feature-specific visual pathways and adjacent audiovisual association areas during “*what*” *vs*. “*where*” auditory attention.

## Introduction

There is increasing evidence of separate auditory-cortex pathways for object and spatial features [1], [2], analogous to the parallel *“what”* and *“where”* visual pathways [3]. Given the existing knowledge of crossmodal connections [4], the auditory *“what”* and *“where”* pathways may separately interact with their visual counterparts at multiple levels [5]–[7]. However, the exact intersections where the auditory and visual dual pathways meet to govern processing still remain unknown, especially when it comes to attentional modulations.

In the spatial domain, attention to auditory or visual locations activates largely overlapping parietal networks [8]–[11] (although some evidence for modality-specific nodes exists [12], [13]). Audiospatial attention systems are often considered subsidiary to visuospatial processes [14]. Indeed, auditory stimuli are more easily mislocalized toward concurrent but spatially incongruent visual events than *vice versa* [15]. However, crossmodal influences in the opposite direction occur as well [16], [17]: Audiospatial attention may govern visual orienting to out-of-view stimuli [11], [18] and improve detection of unexpected visual targets in expected locations of auditory targets [19]. The posterior audiospatial processing stream may also play a critical role in guiding motor and visuomotor processes [11].

Object-centered multisensory attention is less clearly understood. A recent EEG study [20] suggested that attentional control over auditory and visual *“what”* streams is predominantly modality specific. However, sound-object perception can certainly be affected by crossmodal information. For example, visual attention to speakers' lips can modulate perception of ambiguous auditory speech objects [21], and even alter the percepts [22]. Conversely, sounds may affect perception of visual objects [23] and help select relevant events in an environment containing multiple competing visual objects [24]. Recent studies also suggest that conflicting auditory objects may modulate the spread and capture of visual object-related attention across multisensory objects [25], and that attending to either a visual or an auditory object results in a co-activation of the attended stimulus representation in the other modality [26]. Further studies are, thus, needed to elucidate multisensory aspects of spatial *vs*. object-specific attention.

Attention is reflected by modulations in neuronal oscillations, non-invasively measurable with magnetoencephalography (MEG) and EEG. Previous studies suggest that the degree of oscillatory synchronization may tell us whether a spatially confined, local neuronal group is processing an attended stimulus effectively [27], [28]. Different aspects of attentional modulations of brain activity may, however, occur in distinct frequency bands. Neurophysiological studies in the macaque visual cortex, for example, suggest that neurons activated by an attended stimulus show increased synchronization at higher-frequency gamma band (∼35–90 Hz) and decreased synchronization at lower frequency bands (<17 Hz) [27]. Analogous effects have been well documented also in human MEG and EEG studies. That is, increased attentional processing in areas representing task-relevant stimuli has been shown to increase gamma power in human visual [29]–[31], auditory [32]–[34], and somatosensory [35], [36] visual cortices, while increased synchronization at the lower frequency bands, particularly at the alpha range (∼7–13 Hz), has been associated with disengagement of a network representing task-irrelevant stimulus features [37].

Alpha rhythms are a ubiquitous oscillatory phenomenon whose modulations by subjects' alertness and attentional state may be readily observed in the raw MEG/EEG traces even without signal analysis tools. Alpha oscillations increase, for instance, during drowsiness and limited visual input and, conversely, decrease during visual stimulation and tasks [38], [39], which has led to the prevailing interpretation that enhanced alpha activity reflects “idling” [40] or “active inhibition” [41]–[43]. Consistent with this view, when visual attention is strongly focused to one location of visual field, alpha activity may significantly increase in retinotopic visual-cortex areas representing other (*i.e.*, task-irrelevant) aspects of the visual field, possibly reflecting active inhibition of activity in the underlying populations [44]. Such alpha inhibition effects have been shown to correlate with the ability to ignore irrelevant visual stimuli [45]. Not surprisingly, parieto-occipital alpha also increases when auditory [46], [47] or somatosensory [48] instead of visual stimuli are attended. Task-related alpha modulations might, thus, help measure associations between auditory and visual attention networks. Here, we used MEG to study how object *vs.* spatial auditory attention affects cortical alpha oscillations generated in the absence of visual stimuli or tasks.

## Materials and Methods

We reanalyzed a data set, of which different (unimodal, non-oscillatory) aspects of cortical processing have been previously reported [2], to investigate how feature-specific auditory attention modulates oscillatory activity in human visual cortices by utilizing cortically-constrained MEG source estimates.

### Subjects and Design

Nine healthy right-handed (age 21–44 years, 3 females, pre-tested with Edinburgh Test for Handedness) native Finnish speakers with normal hearing participated. During MEG measurements, subjects attended either spatial (*“where”*) or phonetic (*“what”*) attributes of one sound sequence, or ignored stimulation. This sequence included pairs of Finnish vowels /æ/ and /ø/ (duration 300 ms) simulated from straight ahead or 45 degrees to the right (inter-pair interval 3.4 sec, gap between stimuli 250 ms), produced by convolving raw vowel recordings with acoustic impulse responses measured at the ears of a manikin head to approximate free-field stimulation [49]. The sound pairs were identical, phonetically discordant (but spatially identical), or spatially discordant (but phonetically identical). The subjects were instructed to press a button with the right index finger upon hearing two consecutive pairs identical with respect to the target attribute. The target attribute, prompted with a visual cue, alternated in consecutive blocks (60-sec Attend Location and 60-sec Attend Phoneme blocks were interleaved with 30-sec Passive conditions). The subjects were instructed to keep their eyes open and focus on a steady fixation cross.

### Data acquisition

This research was approved by the institutional review board of Massachusetts General Hospital. Human subjects' approval was obtained and voluntary consents were signed before each measurement. Whole-head 306-channel MEG (passband 0.01–172 Hz, sampling rate 600 Hz; Elekta Neuromag Ltd., Helsinki, Finland) was measured in a magnetically shielded room (Imedco AG, Hägendorf, Switzerland). The data were filtered offline to 1–100 Hz passband and downsampled to 300 Hz for subsequent analyses. The electro-oculogram (EOG) was also recorded to monitor eye artifacts. T1-weighted 3D MRIs (TR/TE = 2750/3.9 ms, 1.3×1×1.3 mm^3^, 256×256 matrix) were obtained for combining anatomical and functional data.

### Data analysis

**Figure 1:**
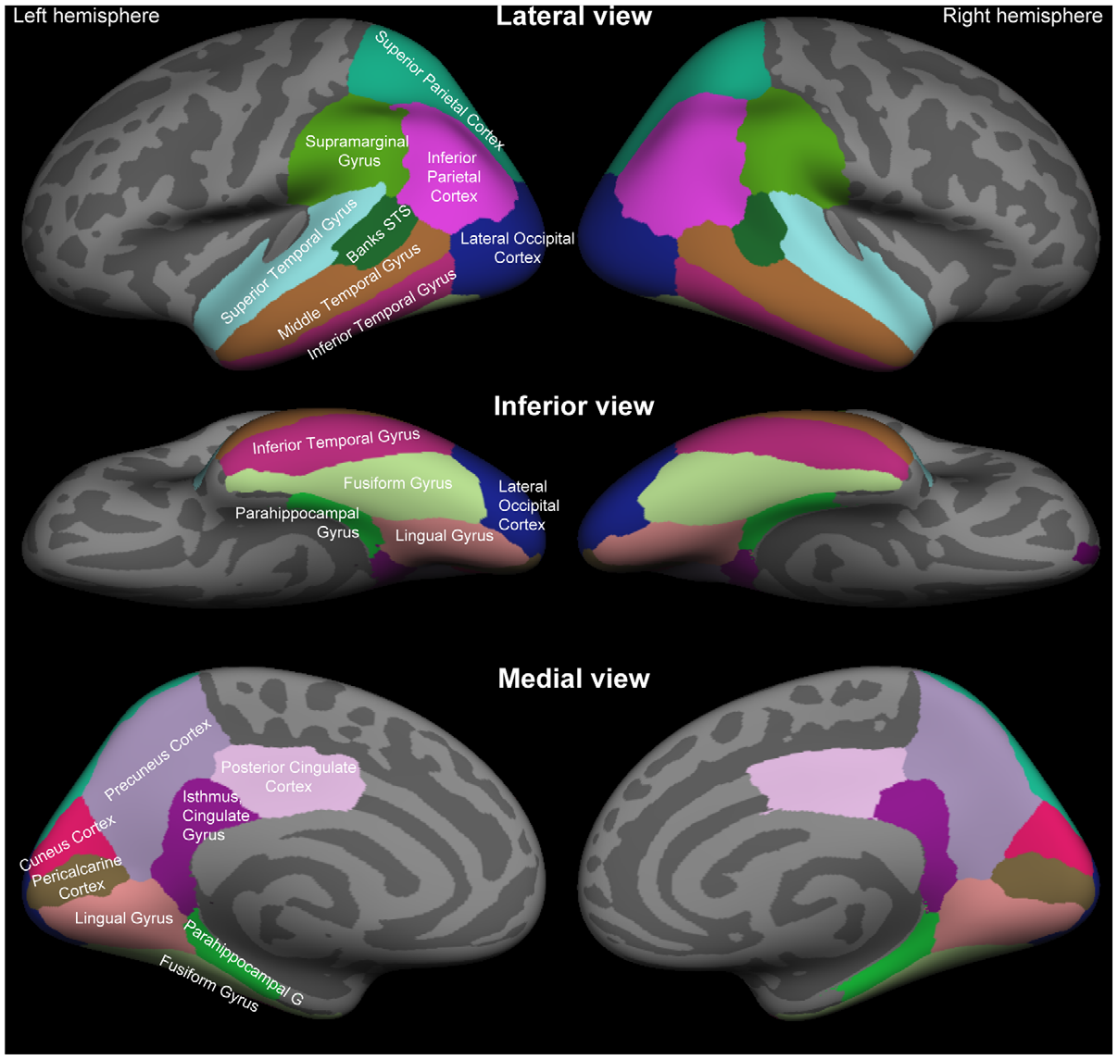
Standard anatomical parcellation of the posterior cortical surface. Color-coded labels of anatomical ROI labels based on the Desikan-Killiany atlas [57] have been shown in the lateral (Top), inferior (Middle), and medial (Bottom) views of the FreeSurfer inflated standard-brain cortical surface. Abbreviation: STS, superior temporal sulcus.

Modulations of cortical oscillatory activity were studied using an MRI-constrained MEG source modeling approach [50], [51]. The information from structural segmentation of the individual MRIs and the MEG sensor locations were used to compute the forward solutions for all source locations using a boundary element model (BEM) [52], [53]. For source estimation from MEG raw data, cortical surfaces extracted [54] with the FreeSurfer software (http://surfer.nmr.mgh.harvard.edu/) were decimated to ∼1,000 vertices per hemisphere. The individual forward solutions for current dipoles placed at these vertices comprised the columns of the gain matrix (**A**). A noise covariance matrix (**C**) was estimated from the raw MEG data. These two matrices, along with the source covariance matrix **R**, were used to calculate the depth-weighted minimum-norm estimate (MNE) inverse operator **W**  =  **RA** ^T^ (**ARA** ^T^ + **C**)^−1^. To estimate cortical oscillatory activity in the cortical sources, the recorded raw MEG time series at the sensors **x(t)** were multiplied by the inverse operator **W** to yield the estimated source activity, as a function of time, on the cortical surface: **s**(**t**)  =  **Wx**(**t**) (*e.g.*, [55], [56]). For whole-brain cortical power estimates, source activities were estimated for all cortical vertices using a loose orientation constraint [55]. Additionally, 16 regions of interest (ROI), selected from areas where crossmodal modulations of posterior alpha activity were hypothesized to be largest, were identified from each subject/hemisphere based on the standard anatomical parcellation of FreeSurfer 5.0 [57] shown in **Figure** **1**. For the TFR analyses, to reduce the computational load, the source component normal to the cortical surface was employed and an average raw data time course was obtained from each ROI, with the signs of the source waveforms aligned on the basis of surface-normal orientations within each ROI to avoid phase cancellations.

**Figure 2:**
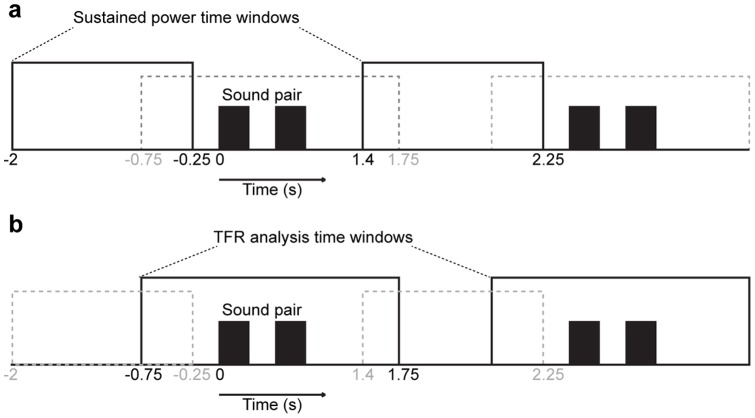
Oscillatory analysis time windows. (**a**) Sustained power analysis time window. Spectral analyses of sustained oscillatory activities were conducted in 1.75 s time windows between sound-pair presentations (solid black rectangles). During this time window, activations driven by the stimuli themselves were assumed to be minimal, while the endogenous processes related to the ongoing selective attention task were presumably strongly activated. (**b**) Analysis window of time-frequency representations (TFR). Dynamic oscillatory power changes were analyzed from a 2.5 s time window overlapping with sound-pair presentations (solid black rectangles). Note that the actual time period for which the power values were obtained is shorter, given the boundary effects in the sliding-window power analysis (*e.g.*, at the lowest frequency of 4 Hz, the effective power time window was −0.38−1.38 s, see **Fig.** **5**).

Oscillatory activity was analyzed using the FieldTrip toolbox (http://www.ru.nl/fcdonders/fieldtrip) [58] and Matlab 7.11 (Mathworks, Natick, MA). To investigate attentional modulations, the data were segmented to epochs with respect to the auditory stimulus-pair presentation, separately for the different attentional conditions. In all analyses, epochs containing over 100 µV peak-to-peak EOG amplitudes were discarded. Sustained/stationary background oscillatory activities were investigated at 4–80 Hz using a fast Fourier transform Hanning taper approach from 1.75 s time windows between sound-pair presentations. This period ranged from 2 s to 250 ms before each sound-pair presentation (**Figure** **2a**), thus constituting a time window by which the sensory-evoked activities to the sound-pairs could be presumed to have ended. After artifact rejection, in the sustained-power analyses, the average number of accepted 1.75-s trials across subjects was 302 during Attend Phoneme, 307 during Attend Location, and 271 during Passive conditions.

For group-level statistical analyses, individual subjects' cortical MNE spectrograms were normalized into a standard brain representation [59]. Group statistical analyses were then conducted within the conventional theta (4–6 Hz), alpha (7–13 Hz), beta (14–34 Hz), and gamma (35–80 Hz) bands. Statistical comparisons of cortical power estimates between the Attend Phoneme and Location conditions were calculated by using a nonparametric cluster-based randomization test [60] (for details, see below). Time-frequency representations (TFR) of dynamic oscillatory changes during and immediately after sound-pair presentations were analyzed from a 2.5 second period starting 0.75 s before the sound-pair onset (**Figure** **2b**). After the artifact rejection, in the TFR analyses, the average number of accepted 2.5-s trials across subjects was 310 during Attend Phoneme, 322 during Attend Location, and 271 during Passive conditions. Subtracting averaged responses from each individual trial before the analyses of spectral power minimized the account of “evoked” stimulus-related processing. The TFR analysis was performed using a fast Fourier transform taper approach with sliding time windows at 4–80 Hz and an adaptive time-window of 3 cycles with a Hanning taper. Power estimates were then averaged over trials. Power TFRs during Attend Phoneme and Location conditions, calculated relative to a pre-stimulus baseline period (*t*<−0.1 s relative to sound-pair onset), were 10×base-10 logarithm normalized for further analyses. An analogous normalization procedure was utilized for ROI analyses of sustained power estimates, which were represented as power values in each active condition, relative to the passive condition.

### Statistical analyses

Statistical significances of differences between the cortical MNE spectrograms were established using a nonparametric randomization test [60]. For cortical power maps, vertices where the *t* statistics exceeded a critical value (two-tail *P*<0.05) of a particular comparison were first identified, and clustered based on their adjacency across the (two-dimensional) cortical sheet (vertex-by-vertex connectivity matrix was determined by scripts from the Brainstorm package, http://neuroimage.usc.edu/brainstorm [61]). The sum of *t* values within a cluster was used as cluster-level statistic, and the cluster with the maximum sum was used as test statistic in the non-parametric randomization procedure [60]. Statistical comparisons of ROI-based TFRs were conducted analogously across the time and frequency: time-frequency bins exceeding the critical value were identified and clustered based on their adjacency across time and frequency, *t*-values sum within time-frequency clusters was used as a cluster-level statistic, the cluster with the maximum sum was used as the test statistic, and, finally, the test statistic for the TFR data was randomized across the two conditions and recalculated 1,500 times to obtain a reference distribution to evaluate the statistic of the actual data. The *a priori* statistical comparisons of means of sustained power estimates in each ROI were established based on t statistics.

## Results

There were no significant differences in reaction times (Attend Location, mean±SEM  = 740±75 ms; Attend Phoneme, mean±SEM = 706±70 ms) between the conditions. However, the hit rate was higher (*F*(1,8) = 28.8, *P*<0.01) in the Attend Phoneme (mean±SEM  = 92±3%) than Attend Location (83±3%) condition. The false alarm rate to “sham targets” (*i.e.*, a phonetic target during Attend Location condition and vice versa; *P* = 12%) was significantly higher (*F*(1,8) = 9.7, *P*<0.05) in Attend Location (mean±SEM  = 5±1%) than Attend Phoneme (1±1%) condition.

### Auditory attention and sustained oscillations in visual pathways

**Figure 3:**
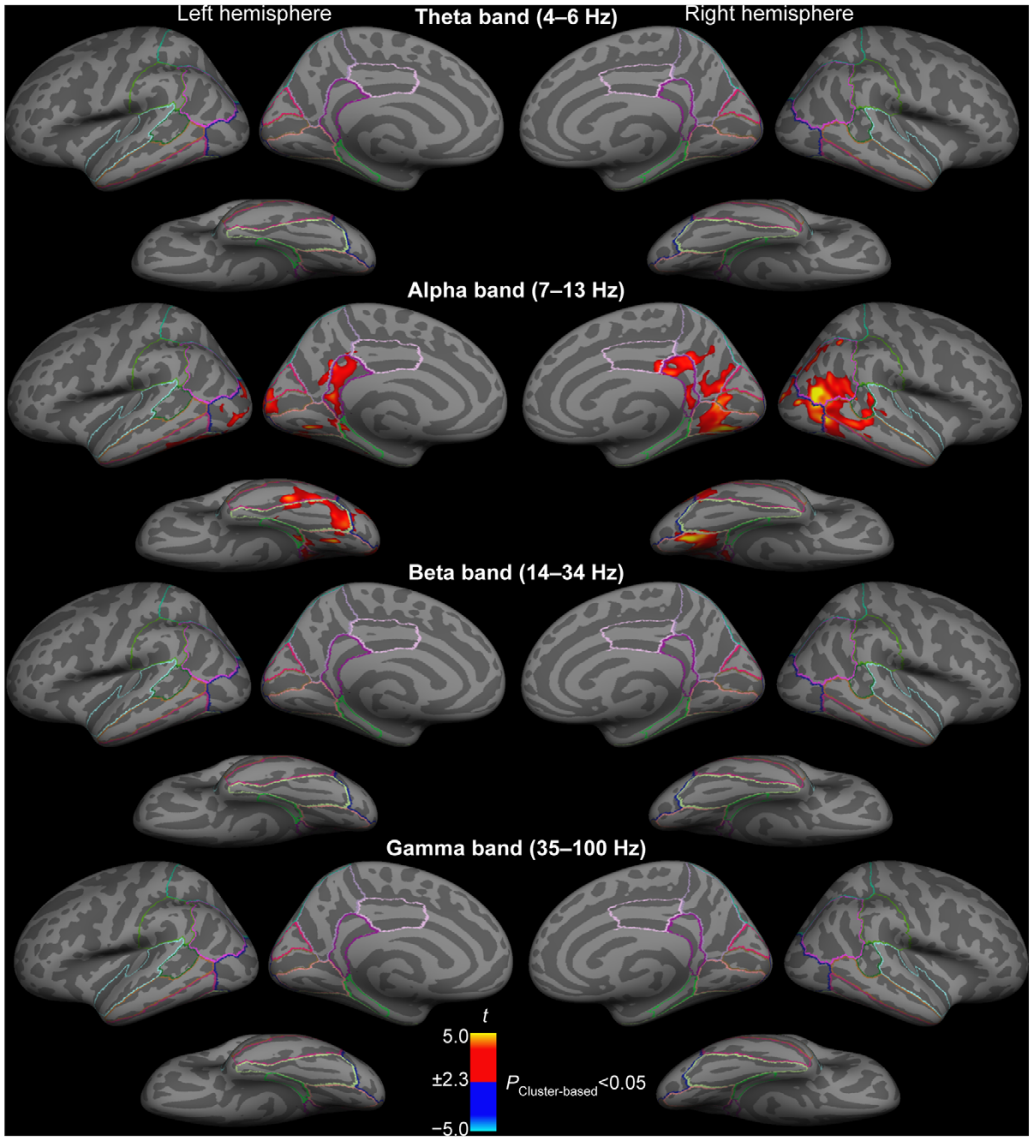
Comparisons of power changes of sustained oscillatory activity between auditory attention to phonetic *vs.* sound location features. The figure shows *t* values masked to locations where the power differences between Attend Phoneme *vs.* Location conditions were statistically significant (*P*<0.05, cluster-based randomization test). For reference, the results have been shown with the outlines of standard anatomical atlas labels specified in detail in Fig. 1. While there were no significant effects at other frequency ranges, the power of background alpha activity was significantly stronger during auditory attention to phonetic than spatial sound features in several visual cortex areas including the primary visual cortex (pericalcarine cortex), left cuneus cortex, lingual gyrus, inferior temporal gyrus, fusiform gyrus, and lateral occipital cortex. Significant increases of alpha activity during auditory phoneme *vs*. location attention were also observed medially in the retrosplenial complex (∼isthmus of cingulate gyrus / precuneus) and precuneus, and laterally in the right inferior parietal cortex, right banks of superior temporal sulcus (STS). In lateral cortex areas, significant alpha increases during phonetic *vs*. spatial auditory attention also emerged near the right-hemispheric area MT (∼near the junction of lateral occipital, inferior parietal, and middle temporal areas).

To examine sustained modulations of visual pathways by auditory attention, oscillatory power changes during periods presumably involving minimal amount of sensory processing related to sound stimuli (1.75 s period starting 1.4 s after the onset of sound pairs) were analyzed. **Figure** **3** shows statistical parameter maps (SPM) of the cortical locations where the Attend Phoneme *vs.* Attend Location conditions were significantly different (*P*<0.05, cluster-based randomization test). To support analysis of anatomical distributions of results, the ROI boundaries, based on the FreeSurfer [57] anatomical atlas, have been superimposed. While there were no significant differences at the theta (4–6 Hz), beta (14–34 Hz), and gamma (35–80 Hz) bands, the sustained alpha (7–13 Hz) power was significantly stronger during Attend Phoneme than Location conditions in widespread areas of posterior cortex. Specifically, significant differences between the two active conditions were observed in parts of the primary visual cortex (pericalcarine cortex), and in the inferior non-primary aspects of the visual cortex, including the lingual, fusiform, and inferior temporal gyri, as well as in the inferior aspects of lateral occipital cortex bordering the fusiform gyrus. Additional significant alpha differences were observed in medial parietal cortices (precuneus) and adjacent retrosplenial complex (∼isthmus of cingulate gyrus). Clusters of significant differences (*P*<0.05, cluster-based randomization test) between the two conditions occurred also laterally in the right hemisphere, extending from the inferior parietal cortices to lateral occipital cortex, medial and inferior temporal gyri, lateral occipital cortex, and superior temporal sulcus (STS). Finally, more superiorly, there were significant differences at the border of inferior and superior parietal cortices, including areas overlapping with the intraparietal sulcus (IPS).

**Figure 4:**
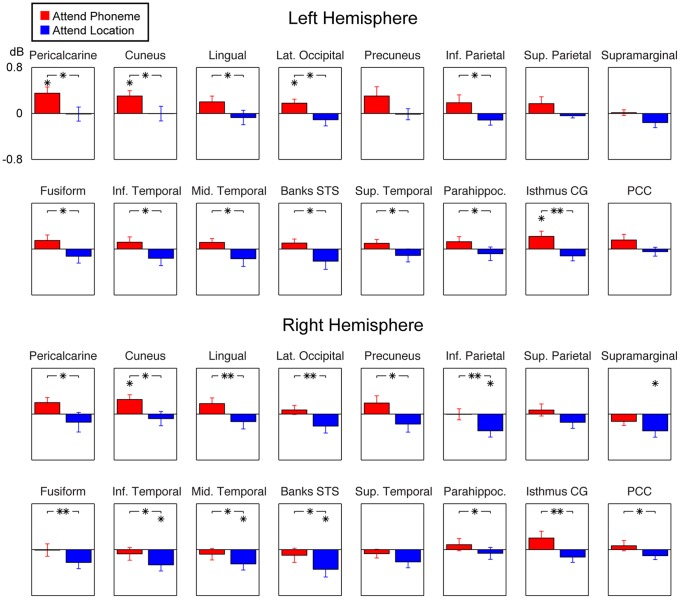
Regions-of-interest (ROI) analyses of alpha activity. The figure shows 10 × base-10 logarithm normalized ROI alpha power estimates during Attend Phoneme and Attend Location conditions, relative to the Passive condition. Consistent with the whole-cortex mapping analyses shown above, these *a priori* comparisons of means suggest significant increases of baseline alpha power in several parietal and occipital ROIs during Attend Phoneme *vs.* Attend Location conditions, indicated by the asterisks with the brackets (*P<0.05, **P<0.01; paired *t* test). In addition to the main comparisons between the two active conditions, statistical comparisons of the 10 × base-10 logarithm normalized relative power (Attend Phoneme or Attend Location relative to Passive) *vs*. zero are also shown, to help determine the polarity of attentional modulations relative to the Passive condition, indicated by the asterisk symbols atop each relevant bar (*P<0.05, *t* test). The normalized amplitude scale is shown in the uppermost left graph.

The results of *a priori* ROI analyses of how different modes of auditory selective attention modulate sustained alpha activities are shown in **Figure** **4**. In these comparisons, measures of active conditions are reported as power values relative to the Passive condition. Consistent with the whole-cortex mapping results, significant differences in alpha power between the Attend Phoneme and Location conditions were observed in the primary and non-primary occipital visual cortices (bilateral pericalcarine, cuneus, lingual gyrus, lateral occipital), inferior temporo-occipital cortex (bilateral fusiform and inferior temporal areas), lateral temporal areas (middle temporal, STS, and left superior temporal areas), parietal cortices (right precuneus, bilateral inferior parietal cortex), retrosplenial regions (bilateral isthmus of cingulate gyrus), right posterior cingulate, and also in the parahippocampal gyri. Although the main emphasis of our analyses were concentrated on comparisons between the two active task conditions (as there was no direct measure of subjects' mental activity during the Passive condition, apart from video monitoring of fixation and EOG measures of blinking activity and eye movements), the results shown in **Figure** **4** also help make inferences of the direction of effects in the two active conditions *vs*. the Passive cognition. Specifically, the polarity can be determined based on the statistical significance of base-10 logarithm normalized relative power *vs.* zero. These analyses suggest that alpha power was significantly larger during Attend Phoneme than Passive condition in the left pericalcarine, bilateral cuneus, left lateral occipital, and in the left isthmus of cingulate gyrus. The differences between Attend Location *vs.* Passive condition were, in turn, lateralized to the right hemisphere, including the inferior parietal, superior parietal, inferior temporal, middle temporal, and STS. Taken together, the general trend of these effects suggest that the main effect shown in **Figure** **3** may be explained by a combination of relative increases alpha during Attend Phoneme and decreases of alpha power during Attend Location condition. However, the alpha increases by phonetic attention were lateralized to the left and the alpha decreases by audiospatial attention to the right hemisphere, with very different spatial distributions.

### Dynamic estimates of oscillatory activity

**Figure 5:**
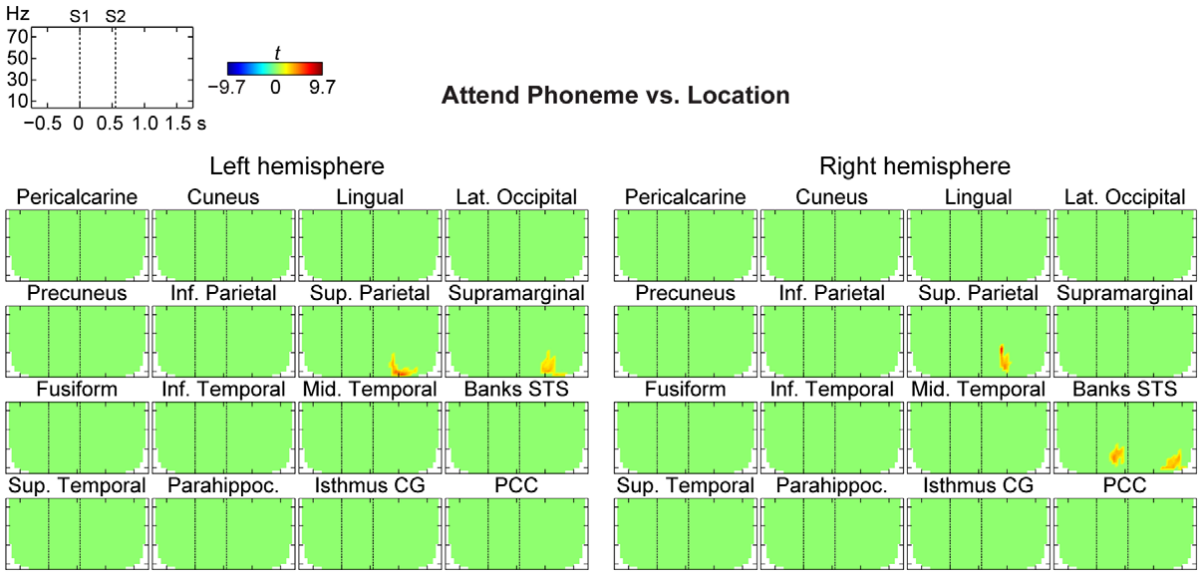
Dynamic time-frequency power analyses of baseline-corrected oscillatory estimates. The figure shows *t* values masked to time-frequency bins where the power differences between Attend Phoneme *vs*. Location conditions were statistically significant (*P*<0.05, cluster-based randomization test). These analyses, from which the account of sustained power changes reported in Figures 2 and 3 have been removed by pre-stimulus baseline correction, transient power changes centered mainly at the alpha range, but also extended to theta and beta ranges, mainly 400–600 ms after the onset of the second sound in the pair (S2).

We then performed TFR analyses of oscillatory activities within a 2.5 second time window around the task-relevant auditory-stimulus pairs (**Figure** **5**). In these estimates, the sustained attentional modulations (reported above in **Figures** **3** **,** **4**) were minimized by using a relative pre-stimulus baseline correction. As shown in **Figure** **5**, there were significant differences (*P*<0.05, cluster-based randomization test) in alpha activity, extending to beta band, between Attend Phoneme and Attend Location conditions, but these differences concentrated mainly in areas beyond the visual sensory areas, including bilateral superior parietal cortices, left supramarginal cortex, and the left STS. In each of these areas, alpha differences centered at around 1 s after the onset of the first sound of the pair (or ∼0.5 s after the onset of the second sound).

## Discussion

The present results demonstrate feature-specific crossmodal influences of auditory attention on alpha activity in posterior visual areas. In comparison to attention to sound location, attention to the identity of sound objects resulted in significant alpha enhancement, probably reflecting reduced processing [40]–[43], in occipital, inferior occipito-temporal, occipital-parietal, and retrosplenial / posterior cingulate gyrus areas. These differences were particularly evident in estimates that were measured from periods between task-relevant stimulus presentation, which were obtained to estimate tonic sustained effects of the different modes of auditory attention on visual system oscillatory activity. While it has been previously known that attending to auditory [46], [47] or somatosensory [48] stimuli may increase visual alpha activity, and suppress visual-cortex fMRI activity in the absence of visual stimulation [62], to our knowledge no previous studies have documented that these effects are dissociable by the feature dimension that is being attended.

### Estimates of sustained oscillatory activities

When subjects attended to the identity of sound objects, and had to ignore the spatial changes in the same stimulus sequence, significant enhancements of alpha power were observed in the right lateral occipito-parietal cortex, including the right lateral occipital cortex, inferior parietal cortex, and posterior STS. These areas have been previously associated with a variety of visual and spatial functions. A highly influential and widely cited theory suggests that these inferior aspects of lateral occipitoparietal cortices and the posterior STS (as a part of the so-called ventral visual attention system) are activated during stimulus-driven capture of visuospatial attention [63]–[66]. Given that during the Attend Phoneme condition subjects needed to actively disregard the concurrently occurring changes in sound direction, it is tempting to speculate that increased alpha power in these areas is somehow reflecting active inhibition of the ventral spatial attention system during auditory phonetic attention. Interestingly, the predominantly right-hemispheric lateral occipital-parietal alpha increases during Attend Phoneme *vs.* Location, which based on the ROI-specific analyses seemed to be explained by significant alpha decreases (that is, increased activation) during Attend Location *vs.* Passive conditions, were consistent with areas where a recent study [67] showed increased activations by auditory “where” *vs.* “what” processing in congenitally blind subjects, suggesting strong connectivity between the posterior audiospatial pathways and these visual cortex areas (which would be expected to be especially enhanced in blind individuals).

Significant differences between Attend Phoneme and Location conditions were also observed bilaterally in the medial parieto-occipital cortices, including the precuneus, and in the adjacent retrosplenial regions. Medial parietal cortices have been shown to be activated during both visual (*e.g.*, [68]) and auditory [8]–[10] spatial attention tasks. As suggested by non-human primate neurophysiological [69] as well as human fMRI [70], [71] and MEG [72] studies, the precuneus is central for complex spatial processes that require combining information from different modalities and spatial frames of references. Such processes include navigation [70], [71], updating object-position information during observer motion [73], and linking motor goals to visuospatial representations [74], [75]. The precuneus has also been suggested to represent the human homologue of the monkey parietal reach region [76], where information of auditory space is converted from head to the gaze centered visuospatial reference frame [77]. One might thus speculate that enhancement of alpha activity in the precuneus during the Attend Phoneme condition follows from active suppression of circuits related to spatial attention and awareness.

Increased alpha power during Attend Phoneme *vs*. Location condition was also observed in the isthmus of cingulate gyrus, which includes the retrosplenial cortex (∼Brodmann Areas 29 and 30) and overlaps with the more broadly defined retrosplenial complex area [78]. Human fMRI studies suggest that retrosplenial are activated during navigational tasks and during passive viewing of navigationally relevant stimuli and spatial memory [70], [71]. Cellular-level neurophysiological studies in rodents have shown that neurons in retrosplenial complex encode spatial quantities, such as head direction [79], [80]. Interestingly, according to tracer studies in the cat, this area has bidirectional connections to the posterior “where” areas of the auditory cortex [81]. Tracer studies in the Mongolian gerbil have shown that ∼10% of all cortical cells with direct projections to the primary auditory cortex are located in the retrosplenial cortex [82], which suggest that this area may also play a role in top-down control of auditory processing. However, it is noteworthy that the medial parietal areas, and particularly the retrosplenial regions, have been associated with many other functions than visual or crossmodal spatial cognition. Further studies are thus needed to determine the functional significance of the present observations.

Finally, in comparison to the Attend Location condition, attention to auditory objects also increased alpha power in ventral occipito-temporal areas, including the lingual gyrus and fusiform cortex. These areas have been traditionally associated with the ventral *“what”* visual pathway [83]. There are two alternative ways to interpret this finding. Assuming that enhanced alpha reflects increased inhibition, it could be speculated that the auditory and visual object processing streams compete against each other. Crossmodal effects consistent with this idea were observed in a recent audiovisual adaptation fMRI experiment [84], showing a coupling between enhancement of supratemporal auditory cortex activities and reductions in visual-cortex *“what”* regions including lateral occipital and fusiform cortices as a function of increasing auditory-stimulus dissimilarity. However, as shown in a recent monkey physiological study [85], the predictions of alpha inhibition theory do not necessarily hold true in inferotemporal cortices, where enhanced alpha power may be associated with increased, not decreased, neuronal firing during selective attention. Applied to the present findings, this would mean that attention to sound identity enhances processing in the inferotemporal visual *“what”* stream. However, this exception of alpha inhibition rule would benefit from further experimental corroboration. More studies are needed to verify the role of increased alpha activity in the ventral “*what*” visual cortex areas during auditory object *vs.* spatial attention.

### Dynamic TFR estimates of oscillatory modulations

The main analyses of the present study focused on sustained oscillatory modulations from time periods between auditory stimuli. The results of these estimates, thus, presumably reflect tonic attentional changes of neuronal activity, related to the sustained engagement of the ongoing attention task. However, the auditory stimuli might also have transiently modulated neuronal activities in the (visual) areas of interest, and an additional dynamic TFR analysis was therefore conducted to compare oscillatory modulations during time windows most likely involving such interactions. These estimates, from which the sustained influences had been removed through baseline normalization, suggested changes that were principally in line with the main analyses of sustained activities. That is, there were brief enhancements of alpha (and low beta) activities during phonetic *vs*. spatial auditory attention in parietal areas and STS after the onset of the second sound of each stimulus pair, possibly reflecting post-stimulus rebounds.

### Potential limitations

The amplitude of alpha oscillations has been shown to correlate with the mental effort required by task performance [86], [87]. It is therefore important to note that in the present study, there were no significant reaction time differences between the task conditions, suggesting that differences, if any, should be small. The observed slightly lower hit rates during spatial attention could suggest that the matching of subsequent sound-location patterns *vs*. phoneme-order patterns might have been more difficult for the subjects (note however that the differences between the directions of 0 *vs.* 45°degrees and differences between the vowels /æ/ and /ø/ were themselves both very easily distinguishable). It is however important to note that this would be expected to result in stronger alpha increases during attention to location, whereas the exact opposite result was observed. On the same note, the task was continuously shifted, at 30–60 second intervals, and it is unlikely that there could have been changes in arousal between the different conditions. It is therefore unlikely that the differences between Attend Phoneme and Attend Location conditions were driven by differences in the level of effort or arousal during the tasks. Another inherent limitation is associated with the lack of objective measure of “ignoring” during the passive listening condition, which complicates the inferences between the active auditory attention and Passive conditions. Therefore, the main statistical inferences in the present study were concentrated on the differences between Attend Phoneme and Location conditions, and the directions of ROI relative amplitude measures have to be interpreted with caution.

MEG source estimation requires appropriate constraints to render the solution unique and regularization to avoid magnification of errors in the process. Our anatomically constrained MNE approach [88] restricts the possible solution to the cerebral gray matter, where a vast majority of recordable MEG activity is generated, to improve the spatial accuracy of source localization. It is also noteworthy that the present effects occurred in pathways that are separated from one another by an order of magnitude larger distance than the previously published MEG source localization accuracy limits [89], [90]. Further, multiple previous studies have successfully differentiated MEG activities originating in the ventral [91] *vs*. dorsal [92] visual streams. Nevertheless, the spatial resolution of present source localization method is not as good as that provided, for example, by fMRI. Meanwhile, finding statistically significant differences between task conditions is, essentially, most probable in areas where the particular oscillatory phenomenon is most predominant, and where the signal-to-noise ratio is best. In other words, a lack of significant modulation of, for example, alpha activity in prefrontal areas associated with either visual or auditory what *vs*. where pathways cannot necessarily be interpreted as contradicting previous findings obtained with other methods, such as fMRI.

### Conclusions

Our data suggest that auditory attention modulates visual processing in a feature-specific manner. In comparison to audiospatial attention, auditory attention to phonetic “*what*” features of sound increased the alpha-band activity in many visual cortex and adjacent association/polysensory areas. In the light of the alpha inhibition theory, relative increases of sustained baseline alpha activity could reflect increased inhibition of the visual system during phonetic *vs*. spatial auditory attention.
